# Monitoring Linked Epidemics: The Case of Tuberculosis and HIV

**DOI:** 10.1371/journal.pone.0008796

**Published:** 2010-01-20

**Authors:** María S. Sánchez, James O. Lloyd-Smith, Wayne M. Getz

**Affiliations:** 1 Department of Environmental Science, Policy and Management, University of California, Berkeley, California, United States of America; 2 Department of Ecology and Evolutionary Biology, University of California Los Angeles, Los Angeles, California, United States of America; National Institute for Infectious Diseases L. Spallanzani, Italy

## Abstract

**Background:**

The tight epidemiological coupling between HIV and its associated opportunistic infections leads to challenges and opportunities for disease surveillance.

**Methodology/Principal Findings:**

We review efforts of WHO and collaborating agencies to track and fight the TB/HIV co-epidemic, and discuss modeling—via mathematical, statistical, and computational approaches—as a means to identify disease indicators designed to integrate data from linked diseases in order to characterize how co-epidemics change in time and space. We present *R*_TB/HIV_, an index comparing changes in TB incidence relative to HIV prevalence, and use it to identify those sub-Saharan African countries with outlier TB/HIV dynamics. *R*_TB/HIV_ can also be used to predict epidemiological trends, investigate the coherency of reported trends, and cross-check the anticipated impact of public health interventions. Identifying the cause(s) responsible for anomalous *R*_TB/HIV_ values can reveal information crucial to the management of public health.

**Conclusions/Significance:**

We frame our suggestions for integrating and analyzing co-epidemic data within the context of global disease monitoring. Used routinely, joint disease indicators such as *R*_TB/HIV_ could greatly enhance the monitoring and evaluation of public health programs.

## Introduction

Epidemiological monitoring presents serious challenges in both developing and developed nations. These challenges can stem from a lack of data, but also from a wealth of data that can be disconnected and analyzed in non-integrative ways. Linked epidemics can offer unique epidemiological insights when comparing interacting disease processes [1], [2], and linked analyses can be particularly insightful if we have a solid understanding of how the epidemics interact. Here we consider analyzing data from closely coupled epidemics—or co-epidemics, as we will refer to them— focusing on the use of joint indicator quantities to integrate data based on known mechanisms of interaction.

HIV offers many dramatic examples of linked emerging and re-emerging epidemics, because it has enhanced the ability of numerous human and zoonotic pathogens to proliferate to unprecedented levels [3], [4]. The epidemiological patterns of several infectious diseases are therefore tightly linked to those of HIV: when the prevalence (total number of persons infected) of HIV increases, so does the incidence (number of new persons with active disease, either because of a new infection, or because an existing infection has become activated or reactivated) and ultimately the prevalence of associated diseases [5], [6]. Tuberculosis (TB) ranks among the most deadly and prevalent re-emerging infections of persons living with HIV/AIDS (PLWHA). In the last 20 years the number of new TB cases has tripled in high HIV prevalence countries, and at least 33% of the world's 33.2 million PLWHA are infected with *Mycobacterium tuberculosis* [7]. Furthermore, drug-resistant TB can be more prevalent and virulent in PLWHA [8]–[10]. There is great regional variation in TB/HIV co-dynamics: approximately 80% of persons with TB/HIV co-infections live in sub-Saharan Africa, where TB is the leading cause of death among PLWHA [7], [11]. The TB burden is expected to increase considerably in Eastern Europe and China, where the HIV epidemic is still rising [12]. Fortunately, because HIV is such a major driving force in HIV associated infections, the incidence and prevalence of opportunistic infections have also been documented to decrease when HIV trends decrease [13], [14].

Here we focus on TB and HIV, reviewing efforts of The World Health Organization (WHO) and collaborative agencies to track and fight the co-epidemic. We discuss the use of mathematical modeling to help identify appropriate disease monitoring indicators, i.e. variables used to measure progress towards the goals, objectives and targets of public health programs [15]. Indicators specifically designed to combine information from both diseases provide an efficient approach to integrating the available data to obtain a more comprehensive view of the entire system, and also facilitate spatiotemporal comparisons of disease dynamics. We present a TB/HIV indicator, *R*_TB/HIV_, which permits us to compare the rate of change of TB incidence relative to that of HIV prevalence, and conduct a comparative study of TB/HIV co-dynamics across sub-Saharan Africa. We also discuss the use of other joint indicators, and conclude by placing our suggestions within the context of global disease monitoring.

## Methods

### Joint Programs for Surveillance and Control of HIV and TB

The tightly knit co-dynamics of HIV and its opportunistic infections have led public health officials to promote and intensify collaborative activities among programs directed toward HIV/AIDS care and control with those focusing on HIV-associated diseases. WHO and collaborators have established a Global TB/HIV Working Group, elaborating frameworks for expanding the scope of TB and HIV programs and their partners [15]–[22] with the objective of improving diagnostic, care, and prevention services for HIV and TB patients. The ultimate goal is to decrease the TB burden in PLWHA and the HIV burden in TB patients [17], [23], [24]. In effect, these efforts have increased the number of TB patients worldwide who were tested for HIV and accessed HIV prevention, treatment, and care services from 22,000 in 2002 to 700,000 in 2006. Additionally, the number of countries implementing collaborative TB/HIV activities rose from 7 in 2003 to 112 in 2006, with Kenya, Malawi, and Rwanda showing exceptional progress. However, much work remains. Many HIV health care facilities are not properly equipped to avoid TB transmission [25] and in 2006, <1% of PLWHA worldwide were tested for TB and only 0.08% were placed on isoniazid preventive therapy when latently infected with TB [26].

Monitoring and evaluation (M&E) is an essential component of health program management [15], and involves the routine tracking of service and program performance (*monitoring*) and the episodic assessment of results of program activities (*evaluation*). As resources devoted to collaborative TB/HIV activities increase, so does the need to assess their quality, effectiveness, coverage, and delivery beyond the tools provided by the guidelines for M&E and lists of indicators established independently by TB and HIV/AIDS programs [15], [17], [27]. For example, monitoring HIV prevalence among TB patients is an activity linked to both programs, and is not exclusive to either one. If neither TB nor HIV/AIDS programs commit funds or accept responsibility for it, key indicators may not be properly surveyed and critical information for adequate M&E may go amiss (10). Likewise, redundancy of research, where both programs monitor the same indicator, should be avoided to conserve limited resources [12]. Accordingly, WHO and the TB/HIV Working Group provide guidance for TB/HIV operational research [12], including a framework for the collection of the most appropriate data, the definition of a core group of indicators, and the allocation of responsibilities specifically targeted at TB/HIV collaborative activities [15]. Furthermore, WHO, UNAIDS and UNICEF provide data on key performance indicators for collaborative TB/HIV activities [11], [16], [28].

### Joint Indicators for Linked Epidemics: The Role of Modeling

Further efforts on behalf of WHO and collaborating agencies in the fight against the TB/HIV co-epidemic include the establishment of research priorities specifically crafted to guide policy development (health system and policy research) and implementation of joint TB/HIV activities (operational research and targeted evaluation) [12]. The ultimate goal of this research is to *improve preventive measures for and care of people with HIV-associated TB*, and is directed primarily toward resource-limited settings in the context of the roll-out of antiretroviral therapy (ARVT) programs. To this end, joint data analysis of TB and HIV co-dynamics via mathematical, statistical, and computational approaches can yield substantial benefits at relatively low financial cost, particularly in the implementation of scaled up or novel public health policies [4], [14], [26], [29]–[33]. Modeling can also offer essential insight in defining optimal indicators, which is a key step in the successful M&E of TB/HIV collaborative activities [15], because analytical techniques can:

- Demonstrate the *purpose* of measuring an indicator by exposing how the different components of the system interact in space and time, potentially revealing hidden inter-relationships between different indicators. Due to the complexity of biological systems, it may not be obvious that certain indicators are surrogate markers for critical population-level disease processes.
- Allow us to *quantify* changes in the value of key indicators in response to disturbances to the system—such as changes in risky behavior, treatment of infected persons, development of drug resistance [31], [32]—thereby facilitating the M&E process by enabling us to contrast expected and reported disease trends.
- Help define the optimal *methodology* and *periodicity* for indicator surveillance. Different surveillance schemes will generate different data types, and modeling can show how informative these types are in regards to particular questions (e.g. via sensitivity analysis, see below). Modeling can also inform on the relative importance of spatial heterogeneity in determining indicator trends [34], allowing us to optimize the spatial scale of data collection. Furthermore, indicators may vary in the amount of information they can provide at different points in time, and modeling can be used to assess the optimal timing of epidemiological surveys.
- Inform on the *strengths* and *limitations* of different indicators by establishing their relative importance via sensitivity and uncertainty analyses [29], [35]. Additionally, these analyses permit us to quantify how our level of uncertainty in the surveillance of an indicator limits our ability to infer actual disease trends from reported trends, or to evaluate the impact of alternative interventions.
- Provide essential insight into spatial and temporal differences in the processes acting on the system [14]. A key attribute of an indicator is its *comparability* across settings, because pandemics such as HIV and TB have a broad geographic distribution, and are subjected to a diverse array of conditions that can generate regional variation in their co-dynamics.

### An Indicator for TB/HIV Dynamics

We can easily quantify how each individual disease varies in time by defining a rate of change of any given measure (i.e. incidence, prevalence, mortality). However, in the case of closely interacting diseases such as TB and HIV, quantifying how the two diseases vary together may provide additional and valuable information both for the individual monitoring of the diseases, and for collaborative control efforts [15], [16]. As such, an essential feature of TB/HIV co-epidemiology is the fact that HIV is the major driver of these diseases' co-dynamics. This occurs because HIV-infected persons are highly prone to develop active TB [36], [37]; in general this risk increases as a person progresses in their HIV infection [38]–[40]. We therefore know how HIV affects TB, and have a general understanding of how the trends of the two diseases are related in time [6], [13], [41]. Hence, a TB/HIV indicator that can capture this causality has the potential to provide greater dynamic insight than a static indicator that provides a one-point-in-time measure. Ideally the dynamic indicator will compare time trends for the two diseases that account for the time lag expected to occur between changes in HIV numbers and changes in TB numbers in a population. By comparing HIV trends from a time period earlier than that considered for TB, we can incorporate this asymmetry in the interaction between the two diseases, where HIV drives TB and not the reverse. As a concrete example of the application of these principles, we present and generalize a measure that was recently applied to HIV and TB co-dynamics in sub-Saharan Africa. This indicator, which we call *R*_TB/HIV_, compares how fast the incidence of active TB cases (including both smear-positive and smear-negative disease) changes in relation to changes in the prevalence of HIV in different countries or regions. We examine changes in TB incidence in the context of varying HIV prevalence—rather than relative changes in the incidence of both diseases or, alternatively, in the prevalence of both diseases—for several reasons. On the one hand, this approach allows us to shorten the time lag between the HIV data and the TB data used for the indicator, because we are capturing the shortest time interval between the measures (HIV prevalence measures people at all stages of their HIV infection, and in principle people that have progressed in their HIV infection will develop active TB disease more rapidly than those only recently infected, which is what is measured by HIV incidence). This increases the total time period over which the indicator can be reliably calculated, because typically we have lower quality, less reliable data the farther we go back in time (i.e., if we compared incidence in both diseases we would need earlier data for HIV). Additionally, HIV incidence and TB prevalence are harder to measure, and their estimation involves a greater number of assumptions, than their counterparts HIV prevalence and TB incidence.

**Figure 1:**
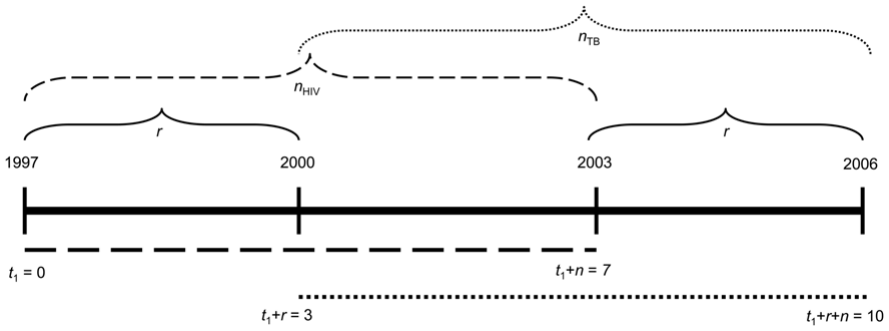
Timeline used to generate 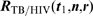 values depicted in Figure 2. The HIV time frame starts at *t*_1_ = 0, corresponding to 1997. In order to track the impact of HIV on TB, we delay the TB time frame by *r* = 3 years. Accordingly, we analyze TB data starting at *t*_1_+*r* = 3 years, i.e. 3 years after 1997, which corresponds to the year 2000. We have TB data until the year 2006, such that the length of our TB time frame spans 2000–2006, for a total of *n* = 7 years of TB data. Under our formulation this corresponds to *t*_1_+*r*+*n* = 10. Because optimally we compare trends for the same number of years for the two diseases, we also use 7 years of HIV data, *t*_1_+*n* = 7 years, i.e. from 1997–2003. Dashed lines indicate HIV, dotted lines indicate TB.

*R*_TB/HIV_ is an indicator that encompasses the ratio of two measures: *R*_TB_, which quantifies the mean change in TB incidence (*I*_TB_) over a defined time period in the context of parameters *t*_1_, *n*, and *r* (see below),
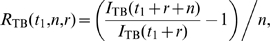
and *R*_HIV_, which quantifies the mean change in HIV prevalence (*P*_HIV_) over an earlier time period,
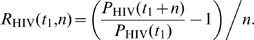

*R*_TB/HIV_ is a modified ratio of these two measures,
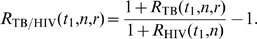
(1)Here *t*_1_ = 0,1,2…, is the index of time progression. The parameter *n* represents the total duration of the HIV prevalence time series. We delay the time frame of the TB data relative to that of the HIV data by *r* years, to account for the average time-lag involved in individuals developing active TB after becoming HIV infected. By doing this, we are essentially designing an indicator measure that incorporates the causal mechanism at play in this epidemiological interaction (see above). To analyze any particular data set it is convenient to set *t*_1_ = 0 as the first year for which HIV prevalence data is available and 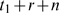 as the last year for which TB incidence data is available, provided HIV prevalence data is available at least until 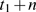 (Fig. 1). Thus, although our indicator is a single number, it evaluates changes in TB incidence relative to HIV prevalence over a period of *n* years, where *n* could be 1 but may be larger if data are collected on a less than annual basis or if averaging over a longer period is desirable (or necessary to smooth the data).

The reason for adding 1 to the rates and then subtracting 1 from their ratio is simply to center the indicator so that it has the intuitive properties listed in Table 1. The sign of the index tells us which disease is increasing or decreasing *relative to the other*. If TB incidence changes at the same rate as HIV prevalence over the period in question, then 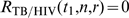. If TB incidence increases relative to HIV prevalence, then 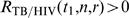. This result can occur either because both diseases increase and TB does so at a faster rate, or because TB increases and HIV decreases, or alternatively because both diseases decrease but TB does so at a slower rate than HIV. The reverse situations will lead to 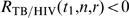, implying that TB incidence decreases relative to HIV prevalence.

**Table 1:** Value of *R*_TB/HIV_ (*R* in the table) as determined by the relative rates of the TB and HIV epidemics.

	HIV increases	HIV constant	HIV decreases
**TB increases**	*R*>0 *if*: TB increase > HIV increase		
	*R* = 0 *if*: TB increase = HIV increase	*R*>0	*R*>0
	*R*<0 *if*: TB increase < HIV increase		
**TB constant**	*R*<0	*R* = 0	*R*>0
**TB decreases**			*R*>0 *if*: TB decrease < HIV decrease
	*R*<0	*R*<0	*R* = 0 *if*: TB decrease = HIV decrease
			*R*<0 *if*: TB decrease > HIV decrease

*R*_TB/HIV_ can be used to analyze the direction of relative change of the two diseases over time in a given geographic area (e.g. town, district, country). However, it has added value because it is a quantity easily comparable across different areas, such as the districts within a country, or different countries within a continent. Therefore one can gain insight into the epidemics' co-dynamics not only because of the numerical value of the measure in a given area, but by comparing its value to neighboring areas (see below). In any case, here we are working with an index of relative growth rate, and consequently from a public health perspective any imbalance generally will be more significant the higher the absolute numbers of TB and HIV for a given region—that is, a given difference in *R*_TB/HIV_ will raise more concerns in badly-affected areas (see below).

## Results and Discussion

### Case Study: TB and HIV in Sub-Saharan Africa

Recently we applied an indicator related to (1) in the course of a study evaluating the potential impact of shortening treatment duration for TB [14], [30], [33]. We focused on high HIV prevalence areas, and used Kenya to calibrate our TB/HIV model because this country provides a cohesive spatial monitoring unit with a sound surveillance record for both diseases. However, our mathematical model could not reconcile reported TB and HIV trends. In an effort to understand TB/HIV co-dynamics in Kenya, we investigated TB/HIV patterns in the whole of Africa by comparing the rates of change of TB as compared to those of HIV [14]. We used a measure related to that shown above, with certain modifications due to data availability. Our analysis singled out Kenya as a clear outlier (together with two other countries), with TB/HIV co-dynamics that were incongruous with the rest of sub-Saharan Africa because of its notable increase in TB trends in relation to HIV trends. Possible explanations for the mismatch included real epidemiological differences or problems with the reported data. The latter appears to have been the case in Kenya, because following the completion of our study the official trends for both diseases have been revised: TB numbers have been revised downwards [42] and HIV numbers are in the process of being revised upwards [43]. That is, in recent years the decrease in HIV was overestimated in Kenya, while improvements in the detection of TB had not been taken into account [14], [42]–[44]. Our joint analysis of TB/HIV data via mathematical modeling and the *R*_TB/HIV_ indicator identified the anomalous co-dynamics rendered by paradoxical TB and HIV data reported for Kenya in 2006 [44]–[47].

**Figure 2:**
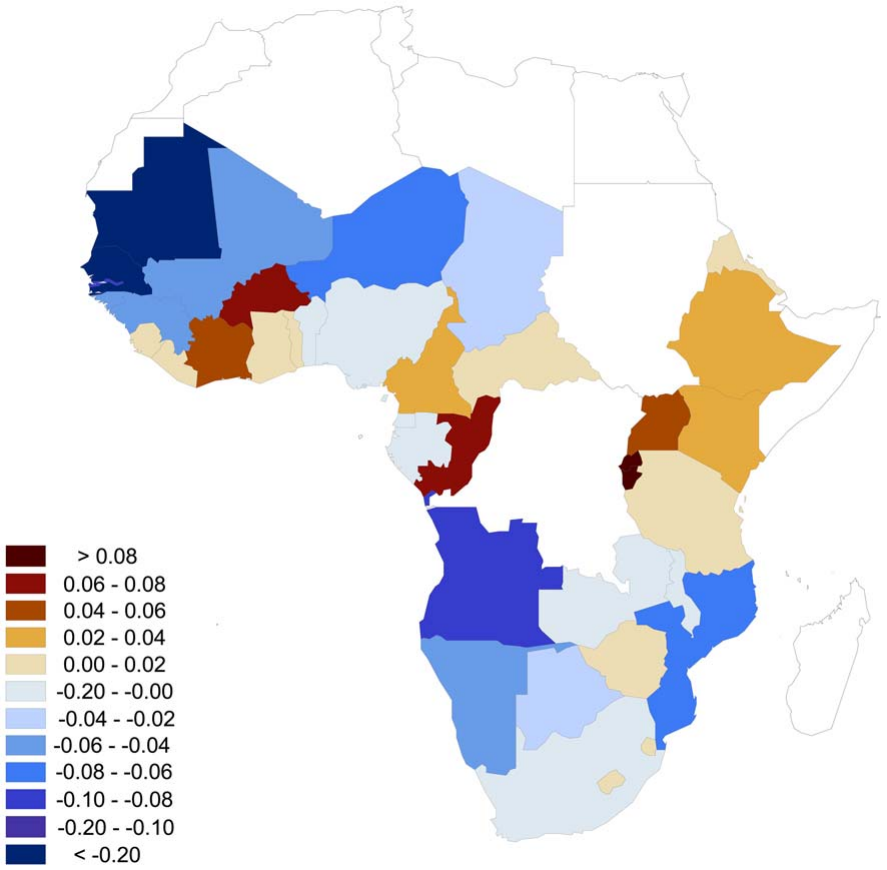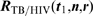 for Africa for the time point *t*_1_ corresponding to the year 1997. Here *n* = 7 and *r* = 3. This index quantifies the change in TB incidence per 100,000 over the period 2000–2006 relative to the change in percent HIV prevalence over the period 1997–2003.

Here we present a re-analysis with the revised TB and HIV numbers. We have calculated the *R*_TB/HIV_ values with the officially reported country estimates for TB incidence [42] and HIV prevalence [43] for Africa (Figs. 1 and 2). We set *r* = 3, i.e. we define the optimal time lag at which to begin tracking potential changes in TB incidence in a population as a response to changes in HIV prevalence as 3 years [38], although *r* = 2 or 4 would yield rather similar results. For HIV in Kenya we are working with preliminary ranges (the final official estimates are not yet available), and have estimated the HIV prevalence as the mid-point of the low and high bounds of the preliminary ranges. As such, TB incidence in Kenya was estimated at 420 in 2000 and 384 in 2006, while the midpoint average HIV prevalence for the same years is 10.6 and 7, respectively. The following calculations give us Kenya's *R*_TB/HIV_ value:
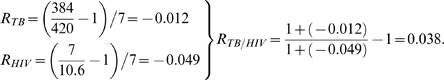
The *R*_TB/HIV_ for all other countries can be calculated with the same basic reasoning. With this re-analysis with the updated data, the incongruency is largely resolved and Kenya's value of *R*_TB/HIV_ ranks 8^th^ out of 38 countries while in our earlier analysis it ranked 3^rd^ [14]. In any case, many sub-Saharan countries are showing a stabilization of their TB incidence rates following a stabilization or decrease of their HIV prevalence trends. The joint TB/HIV trends reported in Africa therefore provide further evidence that substantial benefits follow from decreases in HIV prevalence, in terms of concomitant reductions in HIV-associated opportunistic infections.

Our analysis of TB/HIV co-dynamics in Africa further supports the idea that interacting diseases can be used to monitor each other—an innovative approach to the difficult science of disease surveillance [14]. Analyses of this nature can be used to identify those countries where the reported patterns of linked diseases are incongruent. Looking beyond Kenya, our analysis reveals that TB appears to be outpacing HIV in central Africa, particularly in Rwanda and Burundi (*R*_TB/HIV_ = 0.096 in both countries), while HIV is greatly outpacing TB in northwestern Africa, namely in Mauritania, Senegal, and The Gambia (*R*_TB/HIV_ = −0.318, −0.233, and −0.085, respectively). Once we have identified the countries with outlier co-dynamics, determining the reasons for the imbalance should be a priority because they can reveal information crucial to public health. These reasons may relate to epidemiological mechanisms or surveillance issues or both. For example, if TB is growing disproportionately compared to HIV (indicated by a high value of *R*_TB/HIV_), possible causes could include social conditions linked to poverty, malnutrition and high population density, which can boost TB transmission, or else the spread of more transmissible and virulent TB strains. However, changes in surveillance practices could also contribute or explain the mismatch. Conversely, a disproportionate relative increase in HIV (indicated by low *R*_TB/HIV_ values) may indicate that HIV prevention programs are not effective, and that we can expect to see rising TB numbers in the coming years. *R*_TB/HIV_ can therefore help us predict possible disease trends in the near future.

### Joint Epidemiological Indicators: An Open Field

Studying epidemiological trends of linked infections yields spatial information to include in epidemiological assessments at both small and large spatial scales, spanning regional, country, and district levels. For example, a finer-grained analysis of Kenyan data shows that TB growth in relation to HIV trends is proportionally much higher in the Nairobi district than in the Kisumu district, which could reflect environmental factors favoring TB spread in the high-density settlements around Nairobi [14].

Joint indicators can also be used to investigate the temporal coherency of epidemiological trends from the same area by comparing changes in different time frames from the same localities. We expect the *R*_TB/HIV_ of a specific area or country to vary within certain limits over time; accordingly, this indicator can reveal unexpected co-dynamics with data collected independently by TB and HIV control programs for other purposes. Unexpected *R*_TB/HIV_ values can result, for example, from TB and HIV programs placing greater resources in monitoring their corresponding diseases in different areas at different times, which can yield distorted district and aggregated country estimates. Unequal distribution of public health resources, such as skewed drug delivery programs or imbalanced geographic placement of TB and HIV clinics [34] can also result in differential TB/HIV patterns that can be captured by measures such as *R*_TB/HIV_.

**Figure 3:**
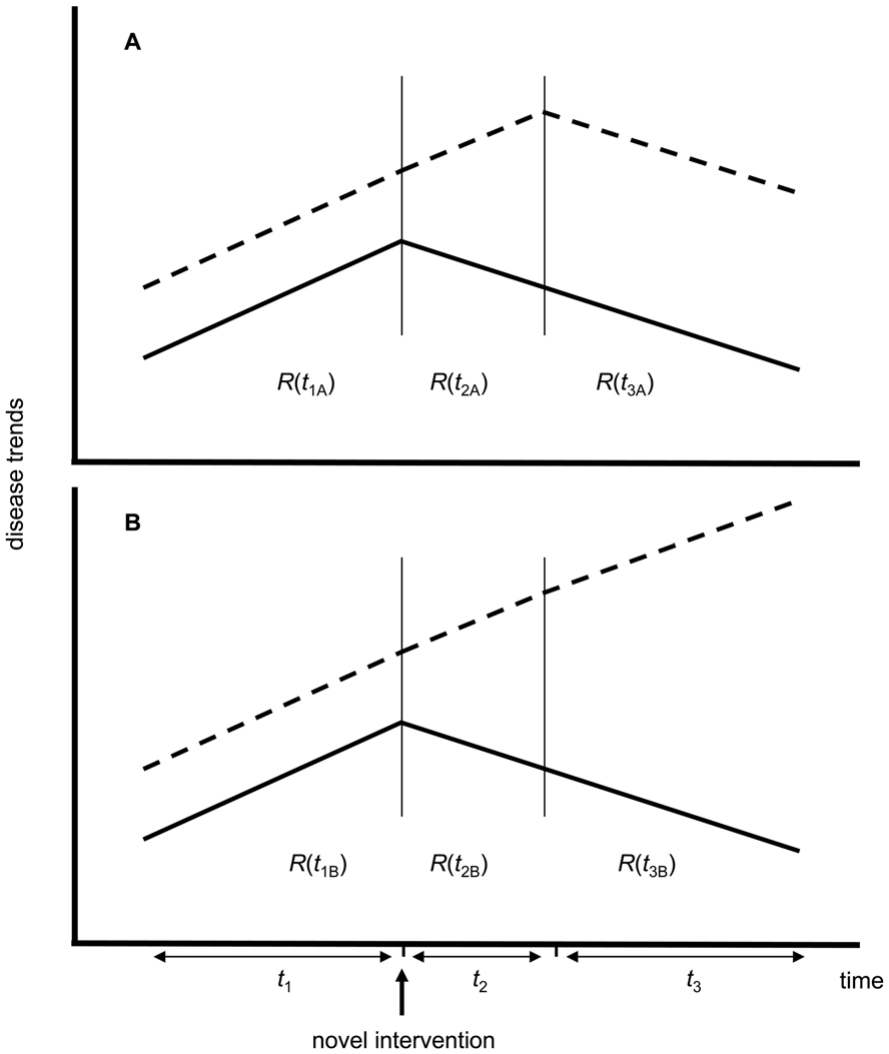
Schematic illustrating the population-level response of the HIV and TB epidemics to a novel HIV control measure. The relative growth rates of percent HIV prevalence (solid line) and TB incidence (dashed line) epidemics before and after the novel intervention (which for example could be the delivery of a new antiretroviral) are captured by 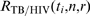 in three different time intervals *i* = 1, 2 and 3. These intervals start before the novel intervention (with *t*_1_ representing the first year for which we have data), when the novel intervention is first implemented (at year *t*_2_), and for the period starting at the point when TB incidence first decreases in scenario A (at year *t*_3_). Years *t*_2_ and *t*_3_ are represented by vertical lines. Because this diagram is a simple schematic we will not define exact values for the parameters *t_i_*, *n* and *r*. While in both scenarios HIV prevalence decreases, in (A) TB incidence decreases after a certain time lag such that (omitting subscripts and common argument values) 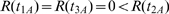, while in (B) TB incidence continues to increase and 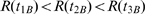, indicating that TB control is not reaping the benefits anticipated from the new HIV control measure.

Another important aspect of the analysis of joint epidemiological indicators is that we can interpret changes to their values in the context of changing control programs for one or both diseases. As an example (Fig. 3), we anticipate that if antiretrovirals are newly introduced into a population, HIV numbers will decrease and in consequence, with a certain time lag, TB numbers will also decrease. Within this predictive scenario, we can compare expected trends with observed trends in order to see if TB control is reaping the benefits expected from the progressive reduction in HIV numbers, and if so by what magnitude, quantified in terms of a joint indicator. By quantifying with a common measure the relative decrease of TB (or any other opportunistic infection) in relation to HIV, we will facilitate comparing the benefits obtained across populations. Comparing data across populations is an excellent research tool that substitutes for ethically unacceptable experimental approaches. As such, anomalous or outlier trends will indicate those localities where benefits are either lower or higher than expected or commonly reported. By identifying these localities we can better investigate which pre-existing conditions, drug delivery systems, ecological settings, or any other relevant factors may maximize TB control benefits in the presence of HIV antiretroviral programs. On the other hand, a reduction in *R*_TB/HIV_ is expected if a scale-up in TB control programs is implemented while HIV programs remained at comparable implementation levels.

Different questions will benefit from defining and using different joint disease indicators. Naturally, we expect there to be a trade off between the complexity of calculating an indicator, the data it requires, the information it provides, and the ease with which it can be adopted by researchers and monitoring agencies. In conjunction, these factors will ultimately determine its usefulness in public health. Easily computed indicators that make intuitive sense are very appealing. However, complex indicators may provide more insight and there use may be warranted in finer analyses—particularly when oddities surface that need to be investigated further. Above we present a relatively simple joint indicator, *R*_TB/HIV_ (1), which is not difficult to compute and is easily understood. However, its comparability *across datasets* is limited because it is not standardized. We can modify *R*_TB/HIV_ by normalizing, thus transforming the measure for each individual country into a dimensionless z-score joint indicator, *Z*_TB/HIV_:
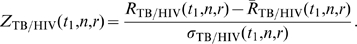
Here *R*_TB/HIV_ corresponds to the measure (1) for a given country, 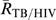 is the mean of all of the individual country *R*_TB/HIV_ for a given dataset, and 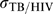 is the standard deviation of *R*_TB/HIV_ for this particular dataset. In the case of the two datasets discussed here—the TB and HIV data for Africa available in [14] and the one available at present, see above—the mean of the first dataset is 0.067 and of the second is −0.015, while the corresponding 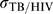 are 0.085 and 0.070. Thus, the *Z*_TB/HIV_ for Kenya is 1.508 in the first dataset and 0.752 in the second (their *R*_TB/HIV_ were 0.195 and 0.038, respectively). Therefore, the updated Kenyan TB and HIV estimates indicate that the relative growth of new TB cases in regards to HIV prevalence is actually approximately half of what was reported in 2006, which reduces the fear that some anomalous conditions are promoting an uncontrollable TB surge in Kenya.

With this example we can see how although *Z*_TB/HIV_ requires us to do further calculations beyond computing all the *R*_TB/HIV_ values for the data of interest, it provides additional insights over these *R*_TB/HIV_ values because it allows us to account for different levels of variability in the data under comparison. When using *R*_TB/HIV_ a direct comparison between the two analyses is complicated by the fact that in [14] we used TB case notifications because of concerns with the reliability of the data, while here we are using the more desirable measure of TB incidence. The values of these two indicators can vary substantially, and in consequence the *R*_TB/HIV_ values also are very different between the two datasets. However, when standardizing with *Z*_TB/HIV_ we facilitate comparing datasets representing different indicators characterized by different units of measurement.

If we needed to investigate questions such as causes of mortality or biases in death reporting systems, we could define indicators that for example compare mortality trends in the interacting diseases, or incidence and mortality trends. Additionally, with the joint indicators *R*_TB/HIV_ and *Z*_TB/HIV_ defined here we are averaging over the time period of choice, and thus will miss any irregularities that occur within the time period. If there are important non-monotonicities in the data such as peaks or troughs, we may benefit from quantifying trend differences not just at the beginning and end of a lengthy time interval, but on a year-to-year (or month-to-month, etc.) comparison. In this case we could average the, for example, year-to-year changes in the trends of both diseases over a certain time period, and then compare the changes in these yearly rates.

As with any other epidemiological measure, the reliability of a joint indicator will be determined by the quality of the surveillance system. We can calculate confidence intervals for our joint indicators that will give an indication of the likely range in which the value of the indicator will fall. Again, however, the reliability of these intervals will be determined by the reliability of the data. In any case, regardless of the statistical complexities we add to our analyses, the main value of using joint indicators for epidemiological monitoring lies in the fact that when contrasting patterns of closely interacting diseases these indices will provide insights into how the diseases are changing with respect to one another over time, in addition to the purely temporal trend information we have of each disease on its own. If the monitoring of one or both diseases is systematically flawed, then anomalous values of the joint indicator will highlight the need for closer scrutiny.

### Looking Forward: The Broader Context

The methodical integration and analysis of epidemiological data for interacting epidemics, both individually and jointly, provides information critical to the design of effective public health measures. The ultimate value of epidemiological indicators, whether for single or linked epidemics, lies in their continued usage over a large proportion of the area covered by an epidemic. The more analyses that are conducted and that can be compared, the more useful an indicator will be. Here we outline key steps in the process of disease monitoring and evaluation, highlighting the use of indicators and analytical techniques relevant to the study of closely linked epidemics. Most of these steps are already being implemented by the corresponding monitoring agencies in many countries and by supra-national agencies.

1. Individual and joint population-level disease indicators must be monitored [15], [16], [48]. It would be valuable to extend this practice to evaluate the co-dynamics of different strains of the linked diseases, particularly regarding drug-resistant strains [4], [31], [32]. Data should be “broken down and reported by the smallest administrative unit possible” [15].
2. Consistent statistical guidelines need to be established for calculating uncertainty ranges for individual and joint measures comparable across all spatial scales.
3. Analyses of long-term temporal trends of key indicators [17] at multiple spatial scales should be conducted using standardized methods in order to ensure the clear communication of analyses and results among monitoring agencies, research groups, and public health personnel. Ideally these analyses should include composite temporal measures as discussed above [14], and graphical and geographic information systems (GIS) analyses whenever applicable.
4. Protocols for acceptable mathematical models need to be developed [49] to analyze past and present trends, and predict future trends. Close collaboration between monitoring agencies and mathematically specialized personnel with training in the dynamics of infectious diseases is strongly recommended.
5. Guidelines should be established for appropriate response to unanticipated epidemiological patterns. These guidelines should identify the personnel of local, national, and supra-national agencies to whom anomalies can be reported, so that further evaluation can take place to identify the underlying cause(s) and prepare suitable response measures.
6. To maximize the utility of data, methods, analyses, and results, information should be disseminated publicly in formats understandable by personnel of different backgrounds [12], [15], [16], [50].

### Conclusions

Advocating, designing, planning and evaluating public health actions rely on sound data analyses [16], [51]. Integrating information from diverse sources can maximize both our understanding of co-infection epidemiology, and our ability to predict accurately the effectiveness of different public health interventions [1]. Accordingly, comprehensive analyses are indispensable for achieving proper integration of TB/HIV collaborative activities, and are critically needed with the on-going scale-up of ARVT programs [12]. Furthermore, M&E of collaborative TB/HIV activities promote a “learning culture” within the programs that guarantees continuous improvement [17]. In our experience, integrating TB and HIV clinical and epidemiological data via a mathematical model, and comparing TB/HIV national trends across Africa via a simple composite measure, permitted us to infer the incongruency of Kenyan TB and HIV estimates. The effort invested in the mathematical model and in the pan-African analysis with the joint co-epidemic indicator, although substantial, was much less than the effort of conducting and evaluating additional surveys. However, the insights provided by our analyses are valuable within the context of TB/HIV global monitoring. Although HIV prevalence among TB patients is probably the most sensitive and reliable indicator for the TB/HIV co-epidemic [17], in our study the relative growth rate of the two diseases, characterized by *R*_TB/HIV_, was even more informative for evaluating Kenyan TB/HIV trends [14]. Importantly for their broad applicability, indices such as *R*_TB/HIV_ can be calculated with the joint analysis of data that are gathered regularly and independently by monitoring agencies. We therefore argue that simple indicators specifically designed to integrate information from closely linked diseases can allow us to gauge how the co-dynamics of epidemics are changing in time, and are powerful tools for spatial comparisons. If used as a routine monitoring tool, these joint indicators could allow public health officials to maximize the use of existing data by evaluating a single number.
